# Indoor Air Quality in Urban and Rural Preschools in Upper Silesia, Poland: Particulate Matter and Carbon Dioxide

**DOI:** 10.3390/ijerph120707697

**Published:** 2015-07-08

**Authors:** Anna Mainka, Elwira Zajusz-Zubek

**Affiliations:** Department of Air Protection, Silesian University of Technology, 22B Konarskiego St., 44-100 Gliwice, Poland; E-Mail: Elwira.Zajusz-Zubek@polsl.pl

**Keywords:** indoor air quality, particulate matter, carbon dioxide, children

## Abstract

Indoor air quality (IAQ) in preschools is an important public health challenge. Particular attention should be paid to younger children, because they are more vulnerable to air pollution than higher grade children and because they spend more time indoors. Among air pollutants, particulate matter (PM) is of the greatest interest mainly due to its acute and chronic effects on children’s health. In addition, carbon dioxide (CO_2_) levels indicate ventilation conditions. In this paper, we present the concentrations of PM (PM_1_, PM_2.5_, PM_10_ and total—TSP) and CO_2_ monitored in four naturally ventilated nursery schools located in the area of Gliwice, Poland. The nursery schools were selected to characterize areas with different degrees of urbanization and traffic densities during the winter season. The results indicate the problem of elevated concentrations of PM inside the examined classrooms, as well as that of high levels of CO_2_ exceeding 1000 ppm in relation to outdoor air. The characteristics of IAQ were significantly different, both in terms of classroom occupation (younger or older children) and of localization (urban or rural). To evaluate the children’s exposure to poor IAQ, indicators based on air quality guidelines were proposed to rank classrooms according to their hazard on the health of children.

## 1. Introduction

The current strategic framework for European cooperation in education and training, assumes that, by the year 2020, at least 95% of children between the ages of four and whenever they start compulsory primary education should participate in early childhood education [1].

In Poland, preschool education covers children aged from three to six years old and is fulfilled by nursery schools, preschool branches in schools and in preschool points. Early childhood education in 48.8% of cases is led by nursery schools, from which 67% are located in urban areas. Preschool education involves 1.3 million children, which covers 75.3% of children. Respectively, in urban and rural areas, 84.4% and 58.6% of children attend preschool education [2]. In Poland, the compulsory participation of five-year-olds results in 93.6% contribution levels for early childhood education. The aim of the EU benchmark to achieve the 95% level will be mainly connected with the increase in attendance of four-year-olds (currently 70.7%) and three-year-olds (currently 57.5%). 

According to the expected development of early childhood education, particular effort should be paid in order to ensure high quality care, and especially to the indoor environmental quality, which is crucial in relation to children’s health and welfare. 

During the past decade, many studies have been conducted to assess IAQ in school environments. A large number of indoor air pollutants have been measured, including volatile organic compounds (VOCs), nitrogen oxides (NO_x_), sulphur dioxide (SO_2_), ozone (O_3_), carbon mon- and dioxide (CO and CO_2_), bioaerosols and PM [3,4,5,6,7,8,9,10,11,12,13,14], but few authors have reported results on IAQ in preschools [15,16,17,18,19,20,21]. Researchers often encounter problems in gaining access to such institutions as nursery schools as well as in installing the necessary measuring equipment in such a way as to avoid disturbance during the measurement process and to limit children’s curiosity.

The exposure of preschool children to air pollutants could represent a very interesting study for three main reasons: (a) children are particularly vulnerable to the harmful effects of air pollution because of immature lung defences, narrower airways, higher inhalation rates and a higher metabolic rate of oxygen consumption per unit of body weight [17,22,23]; (b) younger children spend more time in preschools than in any other indoor environment besides the home; (c) other factors, such as furnishing, sorptive materials (carpets, toys and bedcovers) and children’s activities influence the concentrations of air pollutants, and thus IAQ in preschools is different from primary school onwards [15,17]. 

The present authors’ recent project (“Children’s exposure to indoor air pollutants in nursery schools—CHEIN”) concerning IAQ (VOCs, bioaerosols, PM and CO_2_ concentrations) in urban and rural nursery schools located in the industrial region of Upper Silesia, Poland, clearly indicated the problem of elevated concentrations of PM and CO_2_ during the winter season. 

The Upper Silesia region, compared with other EU countries as well as other Polish regions, is characterized by relatively high levels of PM. A considerable part of the PM comes from anthropogenic sources, such as production processes, coal-based power generation and heat generation industries, as well as traffic and resuspension processes from urban surfaces. However, pollutants emitted from industrial coal combustion processes have been seriously reduced, while the emissions from small-scale combustion utilities, such as domestic boilers, have become particularly dangerous. The hazard of domestic sources originate from low quality of fuels (coal, biomass, culm or even refuse) used for heating during winter. 

The aim of the present study is to characterize IAQ in four nursery school buildings located in urban and rural areas of Gliwice, in southern Poland. The study carried out simultaneous PM (indoor: PM_1_, PM_2.5_, PM_10_ and total—TSP; outdoor: PM_2.5_ and PM_10_) and CO_2_ concentration (outdoors and indoor in two classrooms) measurements to evaluate the influence of outdoor emissions on the IAQ of naturally ventilated classrooms occupied by younger or older children. Moreover, in order to rank classrooms according to their hazard levels, integrated indicators were introduced to correlate WHO guidelines and EU legislation values with the PM_2.5_, PM_10_ and CO_2_ concentrations found in these environments.

## 2. Experimental Section

**Figure 1:**
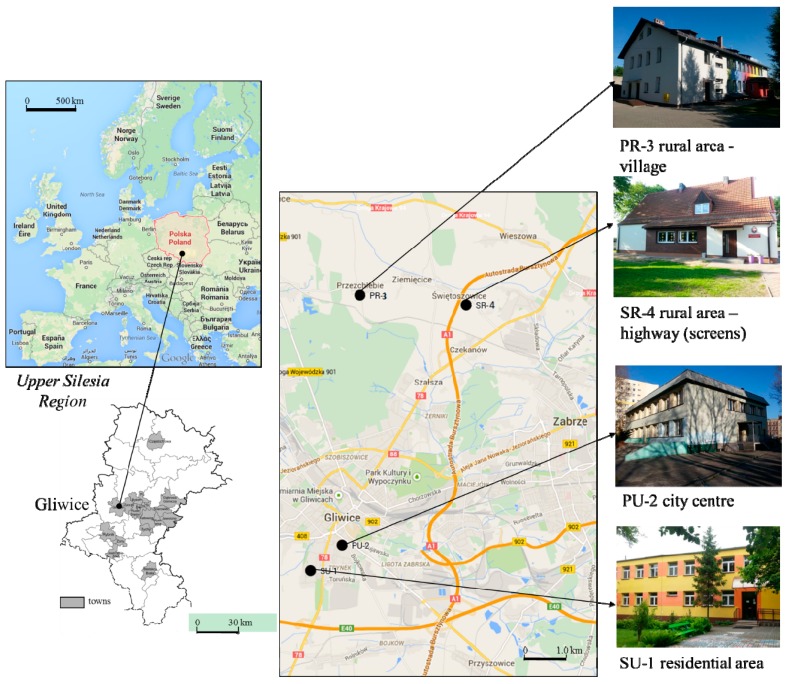
Localization of the investigated nursery schools (Map data: 2015**^©^** Google, ORION-ME).

The study was carried out during the winter season of 2013/2014 at four nursery schools located in the area of Gliwice—a typical city in the industrial region of Upper Silesia, Poland (with 4.5 million people in the region). Two of the nursery schools are located in an urban area and two in a rural area (Figure 1). 

All the nursery schools are public, and entirely managed with public funds by the municipality authorities and the Ministry of Education. 

### 2.1. Sampling Sites

The first building, labelled SU-1 (Sikornik district, Urban area 1), is located in the residential area of the southwest of the city centre, next to an air quality monitoring station. The second is located in an urban traffic area, and labelled PU-2 (Pszczynska Street, Urban area 2). The front facade of the PU-2 building is located 50 m from the street, with heavy traffic reaching 2400–2800 vehicles per hour. Between the PU-2 building and the street, there is parking space available which enables the flow of air from the traffic in the street. Two more buildings are located in the rural areas in the northeast direction approx. 10 km from Gliwice. The location was selected according to the dominant wind direction (207°) in the region. The PR-3 side (Przezchlebie village, Rural area 3) represents typical rural localization without industrial activity or heavy traffic, while the SR-4 building (Świętoszowice village, Rural area 4) is located 50 m from highway A1 (a section opened since 2011). The building is separated from the highway by highway screens. All the nursery schools are located in detached buildings (Figure 1), all of which underwent the process of thermal efficiency improvement, being completed in 2007 (PU-2), 2008 (SU-1) or summer 2013 (PR-3 and SR-4). During the thermal insulation process, the natural ventilation using the air duct systems of the buildings was left unchanged. Consequently, the IAQ is mostly ensured by means of stack ventilation and airing through open and unsealed windows.

The buildings have kitchens which use gas stoves located on the first (SU-1 and SR-4) or ground floors (PU-2 and PR-3). The urban nursery schools had 100 (SU-1) and 150 (PU-2) children divided by age into four or six different classrooms, respectively. Meanwhile, in the rural buildings, 54 (PR-3) and 56 (SR-4) children were divided into three groups. Generally, in each group there were 21–25 children and 1–2 persons on the nursery school staff. In PR-3, there was one group of four disabled children; this group was not included in the studies. The measurements were conducted in older (I) and younger (II) children classrooms. Table 1 summarizes some of the main important characteristics for IAQ in each studied classroom. 

The daytime schedules in all the nursery schools are generally similar. An essential difference between the groups is that the younger children have an afternoon nap from 12:00 to 14:00. In the SU-1 nursery school, children undress, put on pyjamas and - during the resting period—sleep on folding beds covered with their own bedcovers. In other cases, the children rest covered with blankets belonging to the nursery (PU-2) or else themselves (PR-3 and SR-4). During the resting period, they usually watch fairy tales on television - only in PR-3 do the nursery school children rest in another room. 

**Table 1:** Summary of the main characteristics for IAQ analysis in each studied classroom.

Nursery School	Area	Classroom of Children	Children’s Age (Years)	Floor	Volume (m^3^)	Occupancy *	Period of Occupation
SU-1	Urban residential	Older (I)	5–6	Ground floor	180.9	21/25	6:00–17:00
		Younger (II)	3	1st floor	180.9	18/25	8:00–15:00
PU-2	Urban city centre	Older (I)	5–6	1st floor	210.3	20/25	6:30–16:30
		Younger (II)	3	Ground floor	210.3	17/25	6:30–16:30
PR-3	Rural village	Older (I)	5–6	Ground floor	209.2	21/25	6:30–16:00
		Younger (II)	3–4	1st floor	203.7	19/25	8:00–12:30
SR-4	Rural highway	Older (I)	5–6	Ground floor	169.7	16/24	6:30–16:00
		Younger (II)	3–4	Ground floor	118.6	14/21	8:00–12:30

* Median occupancy of children during winter/number of children in group.

### 2.2. Sampling Method

Indoor (PM_1_, PM_2.5_, PM_10_ and TSP) and outdoor (PM_2.5_ and PM_10_) fractions of PM were actively sampled and determined following the reference procedure PN-EN 12341:2014 [24]. Samples were collected between 9 December 2013 and 14 March 2014 both outside on each playground and inside selected classrooms. The sampling time was from Monday to Friday (five-day periods, except for PU-2 (II), which had a four-day sample collection period) between 7:30 and 15:30. Inside the classrooms, the TSP, PM_10_ and PM_2.5_ samples were collected on 25 mm Nuclepore membranes (Whatman International Ltd., Maidstone, UK) while PM_1_ was collected employing 47 mm Teflon filters (Pall International Ltd., New York, NY, USA) using a three-stage impactor with a flow-rate of 30 dm^3^/min. The average volumes of air aspired by the filters was approximately 72 m^3^. Outside, parallel samples of PM_10_ and PM_2.5_ were collected using 47 mm Whatman QMA filters attached to PNS-15 aspirators (Atmoservice, Poznan, Poland) with flow rates of 2.3 m^3^/h. The average volume of air aspired outside by QMA filters was approximately 86 m^3^. Before and after sampling, the membranes and filters were conditioned (temperature 20 ± 1 °C, relative humidity 50 ± 5%) for 48 h and weighted with a microbalance precision of 1 µg. The sampling position in the classrooms was set at the height of an average child’s head (*i.e.*, about 0.8 to 1.0 m above the floor) and away from the door, thus avoiding disturbances resulting from air currents. 

Simultaneously, the continuous measurement of CO_2_ concentrations inside both the (I) and (II) classrooms and outside each building were performed using automatic portable monitors (model 77535, Az Instruments International Ltd., Hong Kong). Each monitor, equipped with a non-dispersive infrared sensor, was connected to a PC with RS232 software installed. The precision of measurements ranged between +0 and +10,000 ppm CO_2_: ±100 ppm CO_2_ or ±3% at a concentration below 100 ppm. The monitors displayed and recorded measurements in real-time, allowing for logged data to be downloaded for analysis. The selected sampling interval was 60 s.

Statistical analyses were performed using the statistical package Statistica 10 (StatSoft, Tulsa, OK, USA). The *t* test for average CO_2_ concentrations (or the Wilcoxon paired sign rank test for the concentrations of PM fractions) was performed in order to test whether urban and rural concentrations differed significantly, as well as whether the concentrations of compounds in older (I) and younger (II) children’s classrooms differed significantly from one another. A statistical significance level of α = 0.05 was used throughout the study.

### 2.3. Integrated Indicators based on WHO Guidelines and EU Legislation

Polish legislation [25] specifies a 24-h mean concentration of PM_10_ in ambient air, which is 50 µg/m^3^. For PM_2.5_, there is no corresponding short-term (24-h) limit, but there is an annual level of 25 µg/m^3^. The World Health Organization (WHO) is more strict in this regard; for PM_2.5_, it recommends a 24-h average standard of 25 µg/m^3^ [26] and recommends applying the same guidelines as for ambient air to indoor spaces.

Carbon dioxide concentrations are often used as a surrogate for the rate of outside supply air per occupant. Permissible concentrations of CO_2_ in confined spaces equal 1,000 ppm. This minimum sanitary requirement is recommended by the European Office of the WHO [27] and by ASHRAE [28]. The Polish Committee for Standardization has established general guidelines concerning the quality of air inside non-residential buildings [29]. This standard was drawn up on the basis of EU directives, as noted in Table 2, through a classification of IAQ, as it is influenced by the CO_2_ concentration above the outdoor level (ΔCO_2_). Based on the above-mentioned regulations, the increase of CO_2_ concentrations in relation to CO_2_ concentrations in outdoor air (ΔCO_2_) was measured in both classrooms of each of the buildings under study.

**Table 2:** Classification of IAQ according to PN-EN 13779 [29].

Category	Description of Indoor Air Quality (IAQ)	Increase of CO_2_ Concentration in Relation to CO_2_ Concentration in Outdoor Air (∆CO_2_, ppm)
IDA 1	High	≤400
IDA 2	Medium	400–600
IDA 3	Moderate	600–1000
IDA 4	Low	≥1000

In order to gain an overall assessment of IAQ in the monitored classrooms, an integrated indicator (Total IAQ_index_) based on recommended guidelines was proposed. The indicator was obtained analogically to the common average air quality index (CAQI) provided to enable a comparison of air quality in European cities [30]. 

The PM index (PM IAQ_index_) was based on the comparison of indoor concentrations with reference concentrations of PM_2.5_ and PM_10_ (WHO guidelines). The PM IAQ_index_ (1) of each fraction was calculated by dividing its average concentration by its corresponding reference concentration, namely 25 and 50 µg/m^3^ for PM_2.5_ and PM_10_, respectively.

(1) $${\text{PM IAQ}}_{\text{index}}=\frac{\text{PM concentration}}{\text{WHO guidelines}}\;$$

The CO_2_ index (CO_2_ IAQ_index_) was created as the fraction of low IAQ (IDA4) during five hours of compulsory preschool education (from 8:00 to 13:00). If the contribution of IDA4 during care/teaching hours was 0%, the CO_2_ index was assumed to be 1.0, and with the increase of IDA4 contribution the CO_2_ IAQ_index_ increased above 1.0:

(2) $${\text{CO}}_{\text{2}}{\text{ IAQ}}_{\text{index}}=\text{1}+\text{IDA4 contribution}$$

The total IAQ index (Total IAQ_index_) was calculated to assess the general exposure risk for each monitored classroom as an average of the IAQ indexes (IAQ_index_) of all the compounds checked at the same sampling side:

(3) $${\text{Total IAQ}}_{\text{index}}=\frac{\sum_{\text{n}}{\text{IAQ}}_{\text{index}}}{\text{n}}$$

where *n* - number of compounds.

## 3. Results and Discussion

The indoor (PM_1_, PM_2.5_, PM_10_ and TSP) and outdoor (PM_2.5_ and PM_10_) concentrations of PM were measured at all the nursery schools. The nursery school buildings differ in location, and so it was expected that they would present different quality outdoor air, especially between the urban and rural sites. At the same time, homogeneous age groups and similar internal sources of air pollutants indicate the validity of performing a comparative analysis between the classrooms of older and younger children, as well as controlling the impact of the afternoon nap on air quality inside the classrooms of younger children.

### 3.1. PM Concentrations

The indoor (PM_1_, PM_2.5_, PM_10_ and TSP) and outdoor (PM_2.5_ and PM_10_) concentrations of PM measured at all the sites are listed in Table 3. As with the indoor samples, outdoor samples of PM_2.5_ and PM_10_ were collected on playground areas. The lowest outdoor PM_2.5_ (21.88 µg/m^3^) and PM_10_ (22.20 µg/m^3^) concentrations were collected on the playground located outside the urban (PU-2) building, while the highest were observed at the playground located at the rural site (PR-3) 88.30 µg/m^3^ (PM_2.5_) and 92.17 µg/m^3^ (PM_10_). The average outdoor PM_2.5_ concentration exceeded the WHO guidelines at 65% and 132%, correspondingly, at urban and rural sites. The average outdoor concentration of PM_10_ collected at the urban sites did not exceed the WHO guidelines, while at rural sites the WHO guidelines were on average exceeded by 30%. The outdoor concentrations were higher in rural areas than in urban areas, but not significantly (*p* = 0.18). Outdoors, during the winter season and in the Upper Silesia region, Rogula-Kozłowska *et al.* [31,32] received analogous mean concentrations of PM_2.5_ and PM_10_. Other worldwide research generally points out higher concentrations of PM at urban sites compared to rural sites [15,17,33]. However, in Poland, during the winter season, domestic sources use low quality coal, biomass or even refuse for heating. Thus, rural areas are often characterized by lower air quality than urban areas.

The highest indoor concentrations of all the determined PM fractions (PM_1_, PM_2.5,_ PM_10_ and TSP) were found in the rural (SR-4) nursery school situated next to the highway. Meanwhile, the lowest were observed in the SU-1 building situated at the urban residential site. However, the discrepancies between urban and rural classrooms are not significant (*p* = 0.10–0.51). The indoor mean concentrations of samples collected in Portuguese preschools were found to be at a similar level, e.g., PM_1_, PM_2.5_, PM_10_ and TSP were 33.08, 34.69, 50.94 and 85.81 µg/m^3^, respectively [17]. Furthermore, the total (TSP) and respirable fraction (PM_10_) of PM in Korean preschools were at a similar level, respectively, 66.5 ± 35.1 µg/m^3^ and 25.9 ± 13.4 µg/m^3^ [15]. Meanwhile, in Swedish preschools equipped with mechanical ventilation, the PM_2.5_ concentrations were significantly lower - between 3.2 and 9.3 µg/m^3^ [21].

**Table 3:** Average levels of PM fractions (μg/m^3^) measured during occupancy periods (*N* = 48); (I) - Older, (II) - Younger children classrooms.

Location	Concentration, μg/m^3^													
	Indoor										Outdoor			
	PM_1_	SD	PM_2.5_	SD	PM_10_	SD	TSP	SD			PM_2.5_	SD	PM_10_	SD
SU-1 (I)	51.21	25.34	70.59	30.71	117.57	42.04	134.43	46.12		35.73		15.49	36.71	16.96
SU-1 (II)	25.97	12.85	41.17	17.91	68.26	24.41	73.05	25.06		42.55		18.45	45.37	20.96
PU-2 (I)	78.89	39.04	106.06	46.14	149.81	53.57	163.81	56.20		64.72		28.07	70.26	32.46
PU-2 (II)	33.70	16.67	49.06	21.34	79.92	28.58	96.78	33.20		21.88		9.49	22.20	10.26
PR-3 (I)	83.64	23.52	102.05	27.54	135.93	41.02	147.54	42.25		44.43		17.21	49.04	17.07
PR-3 (II)	78.13	21.97	80.94	21.84	104.90	31.66	124.24	35.58		88.30		34.20	92.17	32.08
SR-4 (I)	102.11	28.71	125.69	33.92	166.12	50.13	184.24	52.76		38.04		14.74	43.81	15.25
SR-4 (II)	49.04	13.79	67.65	18.26	81.49	24.59	91.19	26.11		60.77		23.54	74.18	25.82
Urban	47.44	23.47	66.72	29.02	103.89	37.15	117.02	40.14		41.22		17.87	43.63	20.16
Rural	78.23	22.00	94.08	25.39	122.11	36.85	136.80	39.17		57.88		22.42	64.80	22.55
(I)	78.96	21.04	101.10	22.81	142.36	20.62	157.50	21.50		45.73		13.19	49.96	14.45
(II)	46.71	23.04	59.70	17.99	83.64	15.35	96.32	21.19		53.37		28.19	58.48	30.93

Moreover, in all the nursery schools, PM concentrations were higher in older children’s classrooms (Table 3). The TSP, PM_10_ and PM_2.5_ levels were significantly higher in older children classrooms (*p* < 0.03), while the PM1 levels were higher but not significantly (*p* = 0.08). Other research reported an analogous relationship between the classrooms of younger and older children [17,33], and indicated a potential reason for this being the cumulative effect of three major conditions: high occupancy, poor ventilation and the intensive activities of children resulting in the PM resuspension phenomenon [7]. 

From our point of view, it was important to explicate which of the enumerated conditions were most influential in the examined cases. Based on Wilcoxon paired sign rank tests, the PM fractions characteristic of older (I) and younger (II) children’s classrooms were compared. In SU-1 and both rural nursery schools (PR-3 and SR-4), the trend of higher PM levels in the older (I) children’s classroom was not as strong or statistically significant (*p* = 0.10–0.48). In the PU-2 nursery school, this seemed more important (*p* = 0.04). 

Assuming that that the physical activity level of five-year-old children is higher than that of three-year-old children in all nursery schools, the process of PM resuspension is able to confirm increased PM concentrations in older children’s classrooms. Moreover, in PU-2, the older children’s classroom was located on the second floor, compared to the first floor in the other nursery schools; this probably increased the influence of the inadequate ventilation system. 

### 3.2. Indoor/Outdoor Ratios and PM Size Distribution

To understand the participation of indoor and outdoor sources in IAQ, the I/O ratios’ analysis was restricted to PM_2.5_ and PM_10_ levels being obtained for each studied classroom (Table 4). The lowest average I/O ratio 0.92 (PM_2.5_) was found in PR-4 (II), occupied by younger children who would leave the classroom for their afternoon nap. Meanwhile, the highest, reaching 3.79 (PM_10_), was in SR-4 (I), occupied by older children. The results underscore the higher contribution of PM_2.5_ and PM_10_ levels in older children’s classroom (respectively 2.30 and 2.97) compared with younger (respectively 1.31 and 1.84) children’s classrooms. In order to appropriately protect the health of children, it is important to reduce their exposure to aerosol particles. For example, instead of the carpets, smooth panels can be installed. In addition, every-day wet cleaning of the floor is strongly recommended. 

In Portuguese preschools, during children’s occupancy, the I/O ratios of PM_10_ varied between 2.12 and 13.96 [17], while the PM_10_ ratios varied from 1.06 to 1.60 in Korea [15]. In one Polish secondary school, during teaching hours in winter, the I/O ratios of PM_1_, PM_2.5_ and PM_10_ were significantly lower at 0.8 ± 0.2, 2.0 ± 0.8 and 2.5 ± 1.7, respectively [6], which might be associated with the reduced physical activity of teenagers.

**Table 4:** Indoor/outdoor (I/O) ratios and PM size distribution in each studied classroom.

Nursery School (Classroom)	I/O Ratios		PM Size Distribution		
	PM_2.5_	PM_10_	PM1/PM_2.5_	PM_2.5_/PM_10_	PM_10_/TSP
SU-1 (I)	1.98	3.20	0.73	0.60	0.87
SU-1 (II)	0.97	1.50	0.63	0.60	0.93
PU-2 (I)	1.64	2.13	0.74	0.71	0.91
PU-2 (II)	2.24	3.60	0.69	0.61	0.83
PR-3 (I)	2.30	2.77	0.82	0.75	0.92
PR-3 (II)	0.92	1.14	0.97	0.77	0.84
SR-4 (I)	3.30	3.79	0.81	0.76	0.90
SR-4 (II)	1.11	1.10	0.72	0.83	0.89

Generally, the I/O ratios were higher for larger fractions of PM_10_ and in all the buildings and classrooms located on the first floor. Blondenau *et al.* [34] have emphasized that the I/O ratios of airborne particles strongly depend on particle size. The larger the particles are in terms of optical diameter, the heavier they are and the more easily they can be deposited on floors and furnishings. Consequently, the influence of re-suspension on indoor particle concentrations increases with particle size. To understand the effect of size distribution on the measured PM concentrations, three different PM size ratios were used to characterize indoor air: PM_1_/PM_2.5_, PM_2.5_/PM_10_ and PM_10_/TSP. The PM size distributions calculated for each classroom generally confirm the predominant contribution of larger particles (Table 4). The relationships between older and younger children’s classrooms were not significant (*p* > 0.33). Larger particles (PM_10_/TSP) at urban and rural sites revealed similar size distributions (*p* = 0.93), while smaller particles presented dissimilar distributions. However, the PM_2.5_/PM_10_ distribution between urban and rural sites was not as significant (*p* = 0.05) as for PM_1_/PM_2.5_ (*p* = 0.004). 

### 3.3. Carbon Dioxide

**Figure 2:**
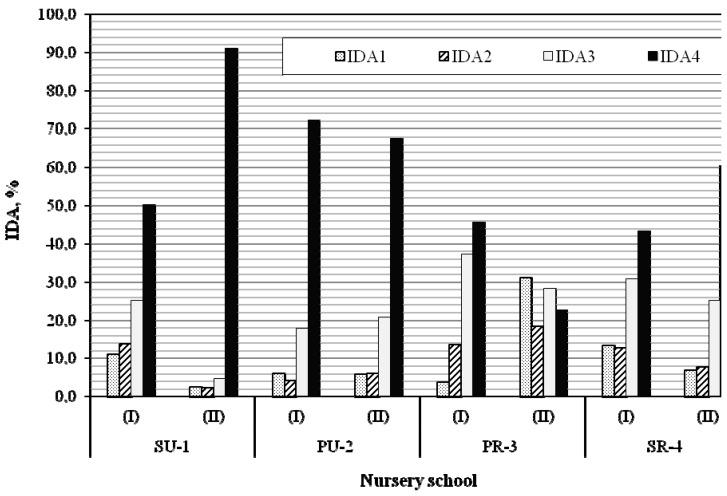
Classification of IAQ (IDA) in nursery school building according to growth of CO_2_ concentration (ΔCO_2_).

Based on the general guidelines concerning the quality of air inside non-residential buildings (PN-EN 13779, 2008), the increase in CO_2_ concentrations in relation to CO_2_ concentration in outdoor air (ΔCO_2_) was measured during the children’s occupation of both classrooms of each of the studied building. Figure 2 depicts the classification of IAQ in each nursery school building during the children’s occupation during compulsory care/teaching hours (8:00–13:00). 

The indoor concentrations of CO_2_ revealed inadequate classroom air exchange rates. Most worryingly, during compulsory care/teaching hours, the air in the classrooms was often of low quality (IDA4). The highest contribution of IDA4 (91.0% of compulsory care/teaching time) prevailed in the younger children’s classroom (II) at the SU-1 site (children who sleep in pyjamas during resting time). Meanwhile, the lowest contribution of poor indoor air (IDA4 = 22.6%) was for the younger children’s classroom (II) at the PR-3 site (children who rest in another room). The differences between indoor and outdoor concentrations of CO_2_ observed in the classrooms of the younger children points to the significance of the afternoon nap.

Recent studies by Gładyszewska-Fiedoruk [18,35] presented CO_2_ levels in nursery schools located in Białystok, in north-eastern Poland. The measurements were taken prior to the arrival of children and staff, as well as after all the classes had been concluded. These studies classified the quality of the air into IDA1 and IDA2 categories prior to the arrival of staff and children, and noted low IAQ (IDA4) in the afternoons (which is in agreement with our own findings). Generally, studies in Poland [35,36,37] and other countries [15,23,38,39,40,41,42] highlight that CO_2_ levels often exceed the recommended standards. Its concentrations vary according to the length and level of occupancy of the classrooms, the type and quality of ventilation and room design. Our research confirmed the enumerated conditions, moreover, our results point out differences between older (I) and younger (II) children’s classrooms, which can become more significant if we link inadequate ventilation with the various patterns of children’s activities.

### 3.4. Integrated Indicator

**Figure 3:**
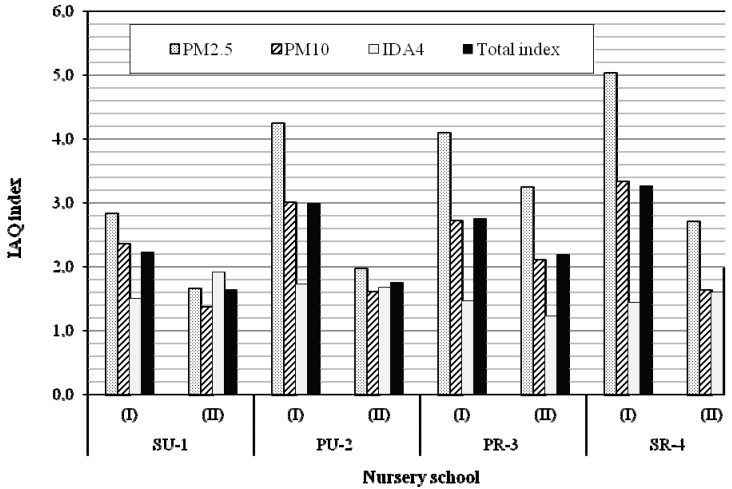
Integrated Indicator -IAQ index.

The IAQ_index_ for each classroom was calculated for PM_2.5_, PM_10_ and CO_2_ (Figure 3). Generally, PM_2.5_ was found to be the highest single-compound contributor to IAQ_index_ values. Only at the urban SU-1 site in the younger children’s classroom (II) CO_2_ IAQ_index_ was higher than PM_2.5_ IAQ_index,_ but not significantly (*p* = 0.43). The individual IAQ_index_ of each compound was assumed to be additive. 

In analogy to the common average air quality index (CAQI) [30], the Total IAQ_index_ was calculated as the arithmetic average of the IAQ_index_ of the individual compounds. Comparing the Total IAQ_index_ for each classroom, the following order of average indicators was found: SR-4(I) > PU-2(I) > PR-3(I) > SU-1(I) > PR-4(II) > SR-3(II) > PU-2(II) > SU-1(II). The presented order clearly indicates the higher indoor exposure of older (five- to six-year-old) than younger (three- to four-year-old) children to the examined air pollutants. The difference in the average Total IAQ_index_ between older (I) and younger (II) children—2.81 and 1.89, respectively—was significant (*p* = 0.01). The urban and rural Total IAQ_index_ were also different - 2.15 and 2.55, respectively - but not significant (*p* = 0.38). 

The key intent of this study was to estimate whether the overall exposure of children to PM and CO_2_ differs between locations of nursery schools. The Total IAQ_index_ enabled to order the examined nursery schools from the highest to the lowest exposure of children to poor IAQ. The following order of average indicators for Total IAQ_index_ was found: SR-4 > PR-3 > PU-2 > SU-1 = 2.62 > 2.47 > 2.37 > 1.93, respectively. It should be underlined that the presented IAQ gradation of nursery schools is adequate only for winter season. More research is necessary to verify whether, during the spring season a similar or reverse relation will occur, which is crucial to the health of children.

## 4. Conclusions

The aim of this study was to assess IAQ in naturally ventilated nursery schools by conducting a PM and CO_2_ measuring campaign. The quantification of indoor (PM_1_, PM_2.5_, PM_10_ and TSP) and outdoor (PM_2.5_ and PM_10_) fractions of PM revealed significant indoor PM contributions. Frequently-monitored, high-levels of CO_2_ exceeding 1,000 ppm in relation to outdoor air (IDA4) also confirmed low IAQ inside classrooms, which points to the low efficiency of ventilation systems. 

The relationship between urban and rural sites revealed elevated concentrations of all PM fractions at rural sites in outdoor as well as indoor air. The IAQ in older and younger children's classrooms confirmed that the classrooms occupied by older children were found to be those with higher PM (PM_1_, PM_2.5_, PM_10_ and TSP) concentrations; meanwhile, the highest CO_2_ concentration was observed in the classrooms of younger children who slept during the afternoon. In addition to the highest contribution of low IAQ during teaching hours, the role of the afternoon nap seems significant.

In order to rank sites based on their IAQ, the total IAQ index (Total IAQ_index_) was used. This simple approach could be a precious tool for the more efficient management of resources, with the aim of mitigating actions, as well as for the estimation of the impact of the indoor activities of children frequenting these environments. 

Similar studies should be conducted in order to evaluate whether or not there is a causal relationship between pollutant exposure and health symptoms in nursery schools and whether this may adversely affect children’s attendance. 
